# Glomerulosclerosis is a prognostic risk factor in patients with membranous nephropathy and non-nephrotic proteinuria

**DOI:** 10.1080/0886022X.2023.2188088

**Published:** 2023-03-26

**Authors:** Jing Sun, Mengfei Li, Qianshen Zhu, Yuanyuan Jia, Jia Tian, Chao Zhang, Xuanyi Du

**Affiliations:** a Department of Nephrology, The Second Affiliated Hospital of Harbin Medical University, Heilongjiang, China; b Department of Nephrology, Wuhan Hospital of Traditional Chinese and Western Medicine, Wuhan, China; c Department of Nephrology, The Fourth Affiliated Hospital of Harbin Medical University, Heilongjiang, China

**Keywords:** Membranous nephropathy, non-nephrotic proteinuria, glomerulosclerosis, risk factor, renal composite endpoint

## Abstract

**Objective:**

To explore the predictive value of the proportion of glomerulosclerosis (GS) incidences on the progression of membranous nephropathy with non-nephrotic proteinuria (NNP).

**Methods:**

This study was a single-center, retrospective, cohort study. Patients with biopsy-proven idiopathic membranous nephropathy were divided into three groups based on the proportion of glomerular sclerosis, and their demographic, clinical, and pathological data were compared. The proportions of primary and secondary endpoints were recorded, and the relationship between GS and primary outcomes (progression to nephrotic syndrome, complete remission, and persistent NNP) and the renal composite endpoint was analyzed.

**Results:**

A total of 112 patients were divided into three groups according to the proportions of glomerulosclerosis. The median follow-up time was 26.5 (13–51) months. There were significant differences in blood pressure (*p* < 0.01), renal interstitial lesions (*p* < 0.0001), and primary endpoints (*p* = 0.005). The survival analysis showed that prognosis was significantly worse in patients with a high proportion of GS than in those patients with a middle and low proportion of GS (*p* < 0.001). The Cox multivariate analysis showed that after adjusting for age, sex, BP, 24-h urinary protein, serum creatinine, treatment scheme, and pathological factors, the risk of renal composite outcome in the low proportion group was 0.076 times higher than that in the high proportion group (*p* = 0.009, HR = 0.076, 95% CI: 0.011–0.532).

**Conclusion:**

A high level of glomerulosclerosis was an independent risk factor for the prognosis of patients with membranous nephropathy with non-nephrotic proteinuria.

## Introduction

Membranous nephropathy (MN) accounts for approximately 20% of adult nephrotic syndrome (NS) [1,2]. Most patients have typical clinical manifestations, including massive proteinuria, hypoalbuminemia, edema, and hyperlipidemia [3–5]. Patients without typical NS and 24-h urinary protein <3.5 g/d are defined as having MN with non-nephrotic proteinuria (NNP). Although previous studies have suggested that these patients generally perform well [6,7], these studies included only small numbers of patients and limited follow-ups. Michelle et al. [8] recruited 108 idiopathic membranous nephropathy (IMN) patients with NNP who were followed up for more than 1 year. They found that 60% of patients with NNP progressed to NS, and the prognosis was similar to that of the classic nephrotic-at-presentation group.

In 1977, Professor Ehrenreich identified glomerulosclerosis (GS) in the renal tissues of patients with MN. Since then, numerous studies have demonstrated that focal segmental glomerulosclerosis (FSGS) disease was a risk factor for poor prognosis in patients with MN [9,10]. Moreover, a Chinese study showed that GS can predict the poor prognosis of primary MN [11]. However, there have been few cohort studies on patients with MN with NNP. The present study sought to explore the predictive value of GS on the progression of IMN with NNP to NS and whether this scenario represented a renal composite endpoint.

## Methods

### Study population

Patients with biopsy-proven IMN who had NNP were recruited from the Second Affiliated Hospital of Harbin Medical University between January 1, 2008, and June 30, 2021. Patients were followed up *via* telephone and the electronic medical record system. The following inclusion criteria were used: (1) biopsy-proven IMN; (2) number of glomeruli under light microscope ≥8; (3) 24-h urinary protein <3.5 g/d at the time of biopsy; (4) complete clinical and pathological data; (5) follow up time >6 months; and (6) age ≥18-years-old. The following exclusion criteria were used: (1) secondary factors, including malignant tumors, rheumatic diseases, lupus erythematous, and hepatitis B or C virus; (2) patient had been treated with hormones and immunosuppressants before renal biopsy; and (3) patients with serious cardiovascular and cerebrovascular diseases, abnormal liver function, and histories of AKI and severe infections.

### Data collection

The demographic variables included age, blood pressure (BP) at biopsy, sex, and edema condition. Laboratory test results included serum creatinine, albumin, globulin, serum uric, and 24-h urinary protein. Additionally, the renal pathology included the degree of the interstitial lesion. Tubular atrophy lesions and glomerular sclerotic lesions were collected from the patient’s medical records. Patients were rechecked every three months. Moreover, patients were followed up by using outpatient reexaminations or telephone. The laboratory test results were recorded at every visit.

### Study design

Each renal pathology report included light microscopy (>8 glomeruli), electron microscopy, and immunofluorescence staining. The results were jointly obtained by two experienced pathologists in our hospital. Patients were grouped based on the proportion of GS, and differences in the clinical and pathological indications were compared among patients in each group. The proportions of each group reaching primary and secondary endpoints were recorded, and the difference in the proportion was analyzed.

### Definition

(1) GS ratio = number of glomeruli with GS + number of glomeruli with FSGS/number of all glomeruli under a light microscope. (2) High pressure without antihypertensive drugs was defined as having a systolic blood pressure (SBP) of ≥140 mmHg and/or diastolic blood pressure (DBP) of ≥90 mmHg [12]. (3) The estimated glomerular filtration rate (eGFR) was calculated by using the Chronic Kidney Disease Epidemiology Collaboration (CKD-EPI) equation [13]. (4) Primary endpoint: (1) complete remission (CR), 24-h urinary protein <0.3 g/d accompanied by normal serum creatinine levels and normal serum albumin concentrations; (2) progression to NS, 24-h urinary protein >3.5 g/d in patients who had NNP; (3) non-nephrotic range proteinuria, 24-h urinary protein >0.3 g/d and <3.5 g/d. (5) Secondary endpoint: (1) reduced eGFR (by >20%) compared with eGFR at the time of biopsy; (2) end-stage renal disease (ESRD), eGFR <15 mL·min-1·(1.73 m2)^−1^; (3) need for renal replacement therapy; (4) all-cause mortality.

### Statistical analysis

Numerical variables with a normal distribution are expressed as the mean ± standard deviation (SD) and were compared by using analysis of variance (ANOVA). Nonparametric variables were expressed as medians (upper quartile, lower quartile) and compared by using the Kruskal–Wallis test. The Bonferroni correction was used to obtain a corrected P value. Categorical variables were expressed as percentages and compared by using the χ^2^ test or Fisher’s exact test. A Pearson’s test or Spearman’s test were used for the correlation analyses. Additionally, the renal event-free survival outcomes were calculated by using the Kaplan–Meier method and log-rank tests. The Cox proportional hazards model was used to determine whether the glomerular sclerosis proportion was an independent risk factor for outcomes. Statistical analyses were performed by using IBM SPSS statistics (version 26.0, Chicago, IL, USA). All P values were two-tailed, and *p* < 0.05 was considered to be statistically significant.

## Results

### Demographic and clinical characteristics

**Figure 1.**
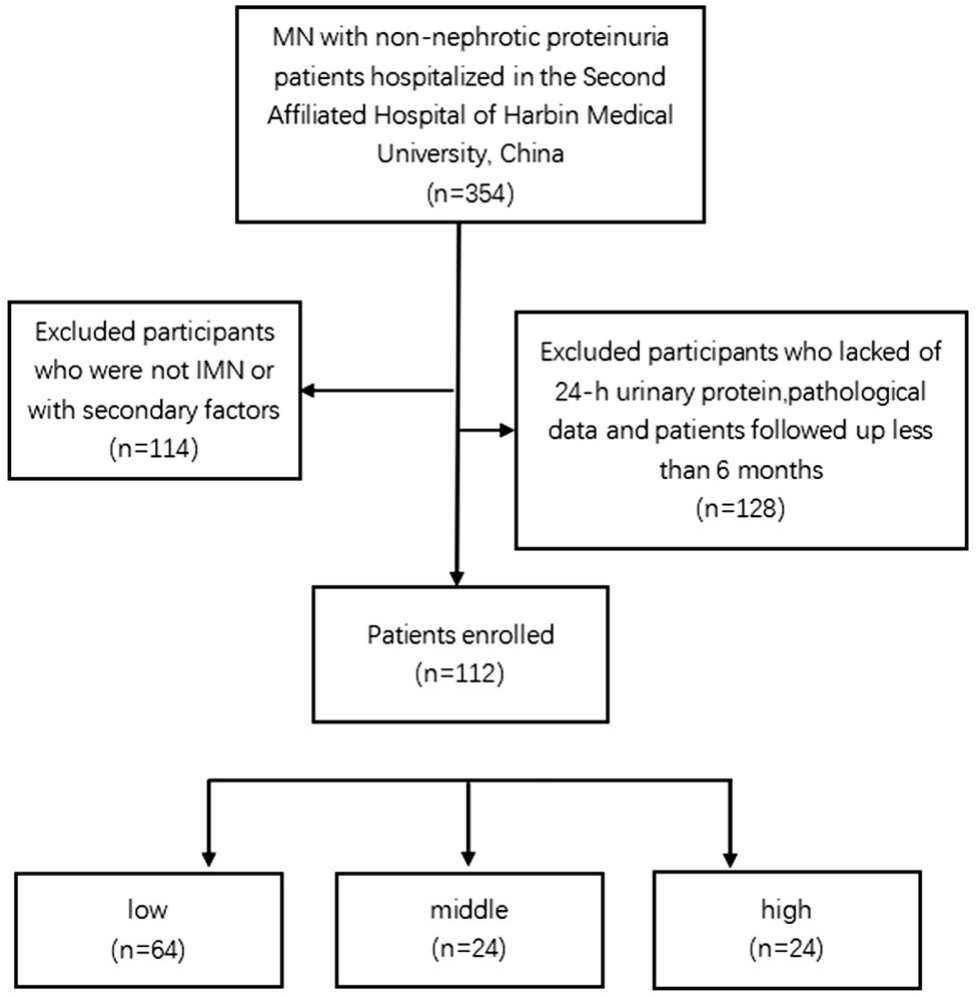
Flowchart of the study.

A total of 112 patients with non-nephrotic range proteinuria IMN were included in this study. According to the proportion of glomerulosclerosis, patients were divided into three groups: low (Group 1, proportion of glomerulosclerosis, 0–0%), middle (Group 2, proportion of glomerulosclerosis, 2.38–7.14%), and high (Group 3, proportion of glomerulosclerosis, 7.69–80%) (Figure 1). The median follow-up time was 26.5 (13–51) months, and men accounted for 44% of the study sample. The average age at diagnosis was 47.45 ± 12.12 years. In total, 86% of patients had edema symptoms, 88% of patients had microscopic hematuria, 2% of patients had negative urine protein, 3% of patients had diabetes, 23% of patients had hypertension, and 67% of patients had hyperlipidemia; moreover, 39%, 53%, and 67% of patients had used ACEI/ARB, corticosteroids, and immunosuppressive agents, respectively. There were differences in SBP, DBP, and hypertension among the three groups (*p* < 0.05) (Table 1).

**Table 1.** Demographic and clinical characteristics.

	ALL (*n* = 112)	Low (*n* = 64)	Middle (*n* = 24)	High (*n* = 24)	*p* Value
**Gender [male (%)]**	48 (42.86)	24 (37.50)	11 (45.8)	13 (54.2)	0.373
**Age (year)**	47.45 ± 12.10	46.53 ± 12.00	46.75 ± 10.26	50.58 ± 13.90	0.360
**Systolic pressure (mmHg)**	128.49 ± 15.62	126.25 ± 14.85	122.92 ± 12.67	140.04 ± 15.08^a,b^	*p* < 0.001
**Diastolic pressure (mmHg)**	82 (76–90)	80 (73.5–86.75)	81 (80–88.5)	88.5 (80–100)^a,b^	0.012
**Edema [*n* (%)]**	95 (84.82)	56 (87.5)	19 (79.2)	20 (83.3)	0.595
**24-h urinary protein (g/d)**	2.21 ± 0.81	2.18 ± 0.82	2.05 ± 0.86	2.45 ± 0.69	0.424
**Serum globulin (g/L)**	25.95 (24.1–28.6)	25.7 (23.95–28.60)	26.05 (24.63–28.33)	26.2 (23–32.3)	0.789
**A/G ratio**	1.20 ± 0.33	1.18 ± 0.32	1.3 ± 0.28	1.16 ± 0.41	0.1885
**Serum albumin (g/L)**	31.06 ± 7.35	30.48 ± 7.19	34.14 ± 6.70	29.53 ± 7.78	0.058
**Serum creatinine (μmol/L)**	61 (53.25–75.75)	59 (51.5–69.25)	62 (56–81.5)	70 (57.5–90)	0.100
**CKD staging [*n* (%)]**					0.230
CKD1	94 (83.93)	55 (85.94)	22 (91.7)	17 (70.8)	
CKD2	15 (13.39)	8 (12.5)	2 (8.3)	5 (20.8)	
CKD3	3 (2.68)	1 (1.56)		2（8.3）	
**eGFR (ml/min/1.73 m^2^)**	102.78 ± 18.48	105.27 ± 18.66	104.27 ± 12.78	94.68 ± 21.04	0.0502
**Serum uric acid (umol/L)**	340.30 (284.95–397.25)	324.6 (275.78–375.13)	342.3 (284.95–415.15)	381.95 (303.53–422)	0.093
**Diabetes [*n* (%)]**	4 (3.57)	2 (3.12)		2 (8.3)	0.389
**Hypertension [*n* (%)]**	25 (22.32)	12 (18.75)	2 (8.3)	11 (45.8)^a,b^	0.004
**Hyperlipidemia [*n* (%)]**	79 (70.54)	47 (73.44)	17 (70.8)	15 (62.5)	0.688
**Coronary heart disease [*n* (%)]**	2 (1.79)	1 (1.56)		1 (4.2)	0.676
**ACEI/ARB [*n* (%)]**	43 (39.39)	20 (31.25)	9 (37.5)	14 (58.3)	0.065
**Glucocorticoid [*n* (%)]**	59 (52.68)	38 (59.37)	10（41.7）	11 (45.8)	0.287
**Immunosuppressant [*n* (%)]**	77 (68.75)	45 (70.31)	16 (66.7)	16 (66.7)	0.924

^a^ *p* < 0.05 Low vs high; ^b^*p* < 0.05 Middle vs high; ^c^*p* < 0.05 Low vs Middle. A/G ratio: Serum albumin/Serum Globulin; CKD staging:Clinical kidney diease staging; eGFR: The estimated glomerular filtration rate; ACEI/ARB: Treatment with angiotensin converting enzyme inhibitors or angiotensin receptor blocker.

### Pathological characteristics

Pathological data of patients, including light microscopy, electron microscopy, and immunofluorescence staining, were collected. A total of 95.54% of the patients were IgG positive, 87% of patients were above 3+, 6.25% of patients were IgA positive, 88.39% of patients were C3 positive, 94.39% of patients were C1Q negative, and 37% of patients were IgM positive. Moreover, sixty-four patients underwent PLA2R staining, of whom 56 patients were positive, with no significant difference. Patients with a high degree of GS had more severe interstitial damage (*p* < 0.0001) (Table 2).

**Table 2.** Pathological characteristics.

	All (*n* = 112)	Low (*n* = 64)	Middle (*n* = 24)	High (*n* = 24)	*p* Value
**IgG**					0.362
–	5 (4.46)	4 (6.25)	1 (4.17)	0	
+-∼++	20 (17.86)	8 (12.5)	3 (12.5)	6 (25)	
+++	87 (77.68)	52 (81.25)	20 (83.33)	18 (75)	
**IgA**					0.879
–	105 (93.75)	60 (93.75)	22 (91.7)	23 (95.8)	
+∼++	7 (6.25)	4 (6.25)	2 (8.3)	1 (4.2)	
**C3**					1.000
–	13 (11.61)	7 (10.94)	3 (12.5)	3 (12.5)	
+-∼+++	99 (88.39)	57 (89.06)	21 (87.5)	21 (87.5)	
**C1Q**					0.371
–	101 (94.39)	58 (93.55)	19 (90.48)	24 (100)	
+	6 (5.61)	4 (6.45)	2 (9.52)		
**IgM**					0.438
–	75 (66.96)	46 (71.88)	14 (58.3)	15 (62.5)	
+∼++	37 (33.04)	18 (28.12)	10 (41.7)	9 (37.5)	
**PLA2R ab**					1.000
–	8 (12.5)	5 (13.16)	1 (11.11)	2 (11.76)	
+∼++	56 (87.5)	33 (86.84)	8 (88.89)	15 (88.24)	
**The pathological stage of MN [n (%)]**					0.085
I	40 (35.7)	26 (40.6)	9 (37.5)	5 (20.8)	
II	55 (49.1)	31 (48.4)	10 (41.7)	14 (58.3)	
III	13 (11.6)	7 (10.9)	2 (8.3)	4 (16.7)	
IV	4 (3.6)		3 (12.5)	1 (4.2)	
**Renal interstitial lesion score**					<0.0001
0	86 (76.78)	56 (87.5)	20 (83.3)	10 (41.7)	
1	24 (21.43)	8 (12.5)	4 (16.7)	12 (50)	
2	2 (1.79)			2 (8.3)	
**Renal tubular atrophy lesion score**					0.117
0	83 (74.11)	52 (81.2)	15 (62.5)	16 (66.7)	
1	26 (23.21)	9 (14.1)	9 (37.5)	8 (33.8)	
2	3 (2.68)	3 (4.7)			
**Number of glomeruli**	14 (10–20)	13 (9–19)	21.5 (17–31)^b, c^	11.5 (10–12)	<0.001
**Percent of renal glomerular sclerosis (%)**	0 (0–6.78)^a, c^	0 (0–0)	5.49 (435–6.67)^b^	15.84 (9.32–24.31)	<0.001

^a^ *p* < 0.05 Low vs high; ^b^*p* < 0.05 Middle vs high; ^c^*p* < 0.05 Low vs Middle; PLA2R ab: Phospholipase A2 receptor antibody; The pathological stage of MN: The pathological stage of Membranous nephropathy under light microscope.

### Prognosis

The patients’ prognoses was followed up, and 66% of patients progressed to NS, and 29% of patients achieved CR. The high GS level group had a higher proportion of progression to NS and a lower proportion of CR. The difference between the three groups was statistically significant (*p* = 0.005). Additionally, the long-term follow-up demonstrated that the high proportion group had more renal outcome events (8/12). The low-proportion group had a better prognosis, and the high-proportion group had a worse prognosis. At the end of the follow-up, there were more patients in the low-proportion group who had achieved CR than in the high-proportion group (Table 3).

**Table 3.** Prognosis.

	All (*n* = 112)	Low (*n* = 64)	Middle (*n* = 21)	High (*n* = 21)	*p* Value
**Primary endpoint**					0.005
CR	29 (25.89)	21 (32.8)	7 (29.2)	1 (4.2)	
NS	66 (58.93)	32 (50)	12 (50)	22 (91.7)^a, b^	
Non-nephrotic range proteinuria	17 (15.18)	11 (17.2)	5 (20.8)	1 (4.2)	
**eGFR decreased by more than 20% [*n* (%)]**	9 (8.04)	3 (4.7)	1 (4.2)	5 (20.8)^a, b^	0.048
**ESRD [*n* (%)]**	1 (0.89)			1 (4.2)	0.429
**Death [*n* (%)]**	2 (1.79)			2 (8.3)	0.089
**NS [*n* (%)]**	12 (12)	5 (8.2)	3 (13.04)	4 (25)	0.161
**Non-nephrotic range proteinuria [*n* (%)]**	47 (47)	27 (44.26)	11 (47.83)	9 (56.25)	0.719
**CR [*n* (%)]**	41 (41)	29 (47.54)	9 (39.13)	3 (18.75)	0.124

^a^ *p* < 0.05 Low vs high; ^b^*p* < 0.05 Middle vs high; ^c^*p* < 0.05 Low vs Middle. CR: complete remission; NS: nephrotic syndrome; ESRD: end-stage renal disease.

### Relationship between the GS ratio and primary and secondary endpoints

**Figure 2.**
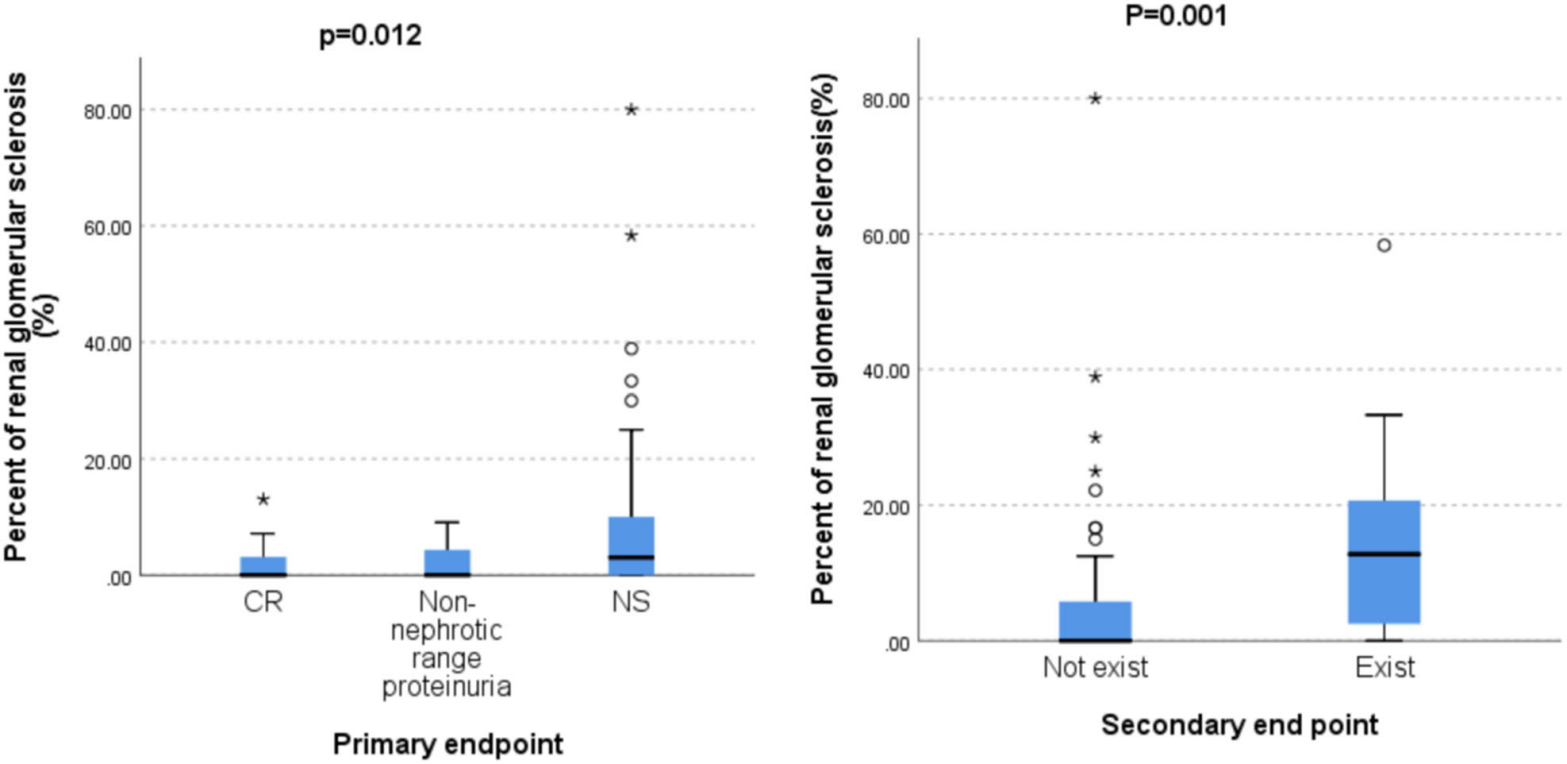
Relationship between glomerulosclerosis and primary and secondary endpoints.

The follow-up data showed that the high proportion group was more likely to develop NS and have the renal composite endpoint. Therefore, the relationship between GS and primary and secondary endpoints was further explored. The level of GS was significantly higher in patients with NS than in those patients with NNP and CR (*p* = 0.012) (Figure 2). The GS rate was significantly higher in patients with renal endpoint events than in those patients without endpoint events (*p* = 0.001).

### Correlation between GS and clinical pathological indices

The correlations between the proportion of GS and demographic, clinical, and pathological data were evaluated. The results showed that the proportion of GS was positively correlated with DBP (*r* = 0.215, *p* = 0.023), pathological stage (*r* = 0.190, *p* = 0.045), the degree of renal interstitial damage (*r* = 0.372, *p* = 0.0001), and renal tubular atrophy lesion score (*r* = 0.198, *p* = 0.036), as well as negatively correlated with eGFR (r = −0.186, *p* = 0.049) (Table 4).

**Table 4.** Correlation between glomerulosclerosis and clinical pathological indices.

	Rho	*p* Value
Gender	−0.109	0.251
Age	0.107	0.262
SBP	0.162	0.089
DBP	0.215	0.023
Edema	−0.036	0.707
Pathological stage	0.190	0.045
24-h urinary protein	0.062	0.517
Serum globulin	0.052	0.588
A/G	0.018	0.854
Serum albumin	0.004	0.967
Serum creatinine	0.177	0.062
CKD stage	0.100	0.295
eGFR	−0.186	0.049
Serum uric acid	0.150	0.116
PLA2R ab	0.023	0.855
IgG	−0.041	0.665
IgA	0.005	0.957
C3	−0.019	0.839
C1Q	−0.068	0.486
IgM	0.139	0.145
Renal interstitial lesion score	0.399	0.00001
Renal tubular atrophy lesion score	0.198	0.036

SBP: Systolic pressure; DBP: Diastolic pressure; A/G: Serum albumin/Serum Globulin; PLA2R ab: Phospholipase A2 receptor antibody; C3: Complement C3; C1Q: Complement C1q; Edema: Edema of the eyelids or the lower limbs.

### Survival analysis

**Figure 3.**
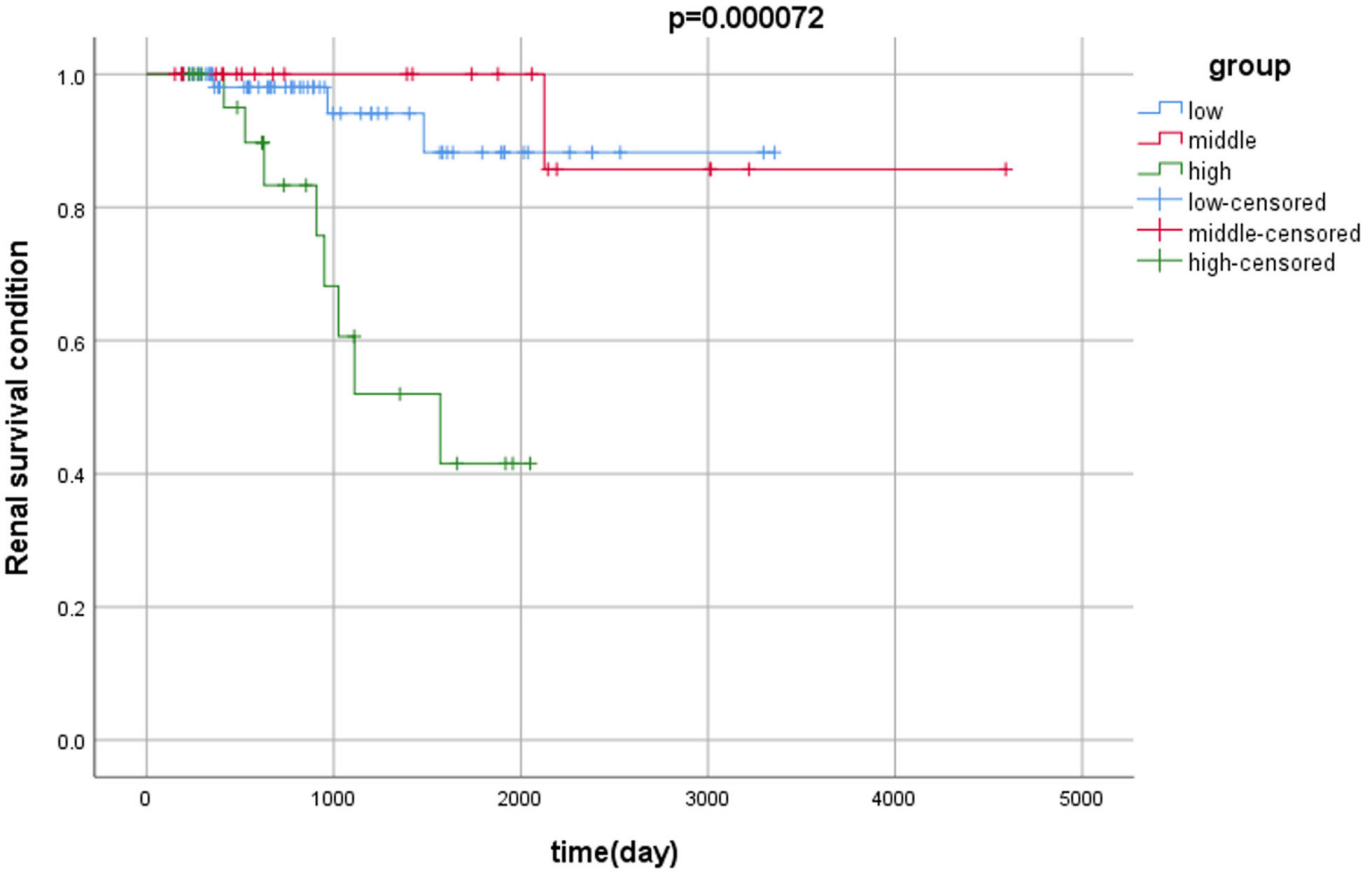
Kaplan–Meier curve survival analysis of patients in the three groups.

Of the 112 subjects, 12 patients reached the renal endpoint after follow-up. The Kaplan–Meier curve survival analysis showed that the survival of patients with a high sclerotic ratio was significantly worse than that of patients with medium and low sclerotic ratios (*p* = 0.00012), but no significant difference was observed between patients with a medium sclerotic ratio and those patients with a low sclerotic ratio (Figure 3).

### Effect of GS ratio on renal prognosis

Cox proportional hazards models were used to further analyze the impact of the GS ratio on the prognosis of patients with MN and with NNP. After correcting for confounding factors, it was found that GS was closely related to the renal prognosis of patients. When other influencing factors were not corrected, the probability of patients reaching the renal composite outcome increased by 3% for every 1% increase in the proportion of GS (*p* = 0.010, HR = 1.031, 95% confidence interval [CI]: 1.007–1.054). After adjusting for age, sex, BP, 24-h urinary protein, and serum creatinine, the probability of patients reaching the renal composite outcome increased by 5% for every 1% increase in the GS rate (*p* = 0.021, HR = 1.056, 95% CI: 1.008–1.105). After the corrective treatment scheme, the probability of patients reaching the renal composite outcome increased by 8% for every 1% increase in the proportion of glomerulosclerosis (*p* = 0.019, HR = 1.085, 95% CI: 1.104–1.161).

A further grouping analysis demonstrated that with unadjusted confounding factors, the risk of renal composite outcome in the low group was 0.057 times higher than that in the high proportion group (*p* = 0.014, HR = 0.057, 95% CI: 0.006–0.562). After corrections for age, sex, BP, 24-h urine protein, and serum creatinine, the risk of renal composite outcome in the low proportion group was 0.127 times higher than that in the high proportion group (*p* = 0.022, HR = 0.043, 95% CI: 0.022–0.0742). After the correction of the treatment plan, the risk of renal composite outcome in the low proportion group was 0.088 times higher than that in the high proportion group. After further corrections for pathological factors, the risk of renal composite outcome in the low proportion group was 0.076 times higher than that in the high proportion group (*p* = 0.009, HR = 0.076, 95% CI: 0.011–0.532).

## Discussion

In recent years, epidemiological studies on MN have shown that its proportion in primary glomerular diseases is increasing [14,15]. In northern and northeastern China, the proportion of MN in primary glomerular diseases has exceeded that of IgA nephropathy, and it has become the most common primary glomerular disease [16]. Primary MN is an autoimmune disease. Some untreated patients can maintain stable renal function and have spontaneous remission. Moreover, MN is a progressive disease, and approximately 20–30% of patients progress to ESRD without treatment. The treatment of MN is very complex; therefore, clinicians are cautious when choosing treatment plans. Previous studies have often only focused on patients with nephrotic proteinuria, whereas studies on patients with MN and with NNP remain scarce. However, for non-nephrotic proteinuria patients, the condition is not serious, and early attention to treatment can often lead to a better prognosis.

GS is a pathological damage that can be divided into spherical sclerosis and segmental sclerosis [17]. It is characterized by decreased glomerular endothelial cells, the accumulation of mesangial matrix, the proliferation of podocytes, and the thickening of the basement membrane. It is an important pathological basis for ESRD and the outcome event of almost all chronic renal disease progression [11]. Previous studies have shown that the deposition of subcutaneous immune complexes on the glomerulus may damage the attachment of podocytes and the basement membrane, and the exfoliation of epithelial cells and the basement membrane may lead to GS [9]. The present study found that IMN patients with a high proportion of GS were more likely to have hypertension, which is consistent with the results of previous studies [18,19]. Li et al. [19] examined 305 IMN patients and found that the incidence of hypertension was higher in the group with FSGS than in the group without FSGS (75.5% vs. 46.0%, respectively, *p* < 0.005), and the average SBP and DBP measurements were higher in the group with FSGS. Dumoulin et al. [9] showed that the only statistically significant indicator was the difference between hypertensive IMN patients with FSGS and those patients without FSGS, and the incidence of FSGS in hypertensive IMN patients was higher. Our study also found that a higher proportion of glomerulosclerosis corresponded to more serious damage to the renal interstitium, which was also confirmed by Wei et al. [11].

The current study found that a higher proportion of glomerulosclerosis corresponded to a worsened prognosis of patients. Wei et al. [11] assessed 200 IMN patients and demonstrated that patients with IMN with a GS ratio ≥6.45% often had poor basic renal function and a poor prognosis. Moreover, a higher proportion of GS in patients corresponded to a greater probability of renal end-point events. Li et al. [19] investigated 305 IMN patients and concluded that FSGS is a risk factor for IMN disease progression. However, some studies have noted that GS is related to the patient’s age, sex, BP, and glomerular filtration rate. After adjusting for these clinical parameters, the guiding significance of combining FSGS for renal prognosis was very limited. Thus, GS still has guiding significance for the prognosis of IMN patients with NNP IMN by establishing Cox proportional hazards models and by correcting for relevant parameters.

In addition, we found that interstitial fibrosis and tubular atrophy lesions may also be associated with renal prognosis. Patients with a high degree of GS had more severe interstitial damage (*p* < 0.0001) (Table 2). The correlation analysis showed that the proportion of GS was positively correlated with the degree of renal interstitial damage (*r* = 0.372, *p* = 0.0001) and renal tubular atrophy lesion score (*r* = 0.198, *p* = 0.036) (Table 4). The Cox regression analysis showed that after further corrections for pathological factors, the GS ratio was not related to the renal prognosis of patients (*p* = 0.107) (Table 5). Therefore, GS, renal interstitial fibrosis, and renal tubular atrophy may jointly affect renal prognosis.

**Table 5.** Cox regression analysis of the glomerulosclerosis ratio and renal prognosis.

Group	Model 0		Model 1	
	P	HR 95% CI	*p* Value	HR 95% CI
	0.010	1.031 (1.007–1.054)	0.021	1.056 (1.008–1.105)
High	Ref		Ref	
Low	0.002	0.111 (0.027–0.452)	0.022	0.127 (0.022–0.742)
Middle	0.014	0.057 (0.006–0.562)	0.028	0.043 (0.003–0.708)
	Model 2		Model 3	
	P	HR 95% CI	*p* Value	HR 95% CI
	0.019	1.085 (1.104–1.161)	0.107	1.024 (0.995–1.053)
Group				
High	Ref		Ref	
Low	0.017	0.088 (0.012–0.652)	0.009	0.076 (0.011–0.532)
Middle	0.007	0.006 (0.0001–0.024)	0.007	0.004 (0.00008–0.226)

Model 0: Unadjusted; Model 1: Adjusted for age, gender, systolic blood pressure, 24-h urinary protein, serum creatinine; Model 2: Adjusted for model 1 plus treatment plan; Model 3: Adjusted for mode 2 plus renal interstitial lesion score and renal tubular atrophy lesion score; CI: confidence interval; HR: hazard ratio; Ref: Reference.

MN with NNP is a special type of MN. Previous studies have shown that MN patients with NNP often have a good prognosis; however, those patients who progress to NS in the later course of the disease have a poor prognosis. A previous study in South Korea showed that low baseline serum albumin was a risk factor for progression to NS [7]. Natural remission mostly occurred in patients with low baseline albuminuria, low serum creatinine, and high serum albumin. However, the study had a short follow-up time period and did not analyze the pathological data of patients. Moreover, our study found that GS is a risk factor for MN patients with NNP that progresses to NS.

### Study limitations and prospects

This study had several shortcomings. First, this was a single-center study with a small sample size. FSGS and GS were not differentiated to evaluate their impacts on the prognosis of kidney disease. Second, this was a retrospective study, and there may be deviations in data collection and the follow-up of patients. Third, there was no consistent standard for the treatment plan of patients, and the choice of clinical treatment was based on the personal preference of the attending physician. Finally, this study failed to clarify the guiding role of the GS ratio in patients’ choices of treatment plan. Future studies should be conducted with a larger sample size to explore the guiding role of the proportion of GS in the treatment of patients.
